# Involvement of SIRT1-mediated aging in liver diseases

**DOI:** 10.3389/fcell.2025.1548015

**Published:** 2025-02-20

**Authors:** Yueming Zhang, Chang Gong, Lina Tao, Jinghui Zhai, Fengwei Huang, Sixi Zhang

**Affiliations:** ^1^ Department of Clinical Pharmacy, The First Hospital of Jilin University, Changchun, China; ^2^ Department of Pharmacy, The First Hospital of Jilin University, Changchun, China; ^3^ College of Pharmacy, Jilin University, Changchun, Jilin, China

**Keywords:** SIRT1, aging, cellular senescence, liver diseases, therapeutic approaches

## Abstract

Liver disease is a significant global health issue, responsible for millions of deaths annually. Aging, characterized by the gradual decline in cellular and physiological functions, impairs tissue regeneration, increases susceptibility to liver diseases, and leads to a decline in liver health. Silent information regulator 1 (SIRT1), a NAD⁺-dependent deacetylase, has emerged as a pivotal factor in modulating age-related changes in the liver. SIRT1 preserves liver function by regulating essential aging-related pathways, including telomere maintenance, epigenetic modifications, cellular senescence, intercellular communication, inflammation, and mitochondrial function. Notably, SIRT1 levels naturally decline with age, contributing to liver disease progression and increased vulnerability to injury. This review summarizes the regulatory role of SIRT1 in aging and its impact on liver diseases such as liver fibrosis, alcoholic associated liver disease (ALD), metabolic dysfunction-associated steatotic liver disease (MASLD), and metabolic dysfunction-associated steatohepatitis (MASH), hepatocellular carcinoma (HCC). We also discuss emerging therapeutic approaches, including SIRT1 activators, gene therapy, and nutritional interventions, which are evaluated for their potential to restore SIRT1 function and mitigate liver disease progression. Finally, we highlight future research directions to optimize SIRT1-targeted therapies for clinical applications in age-related liver conditions.

## Graphical Abstract

**Figure:**
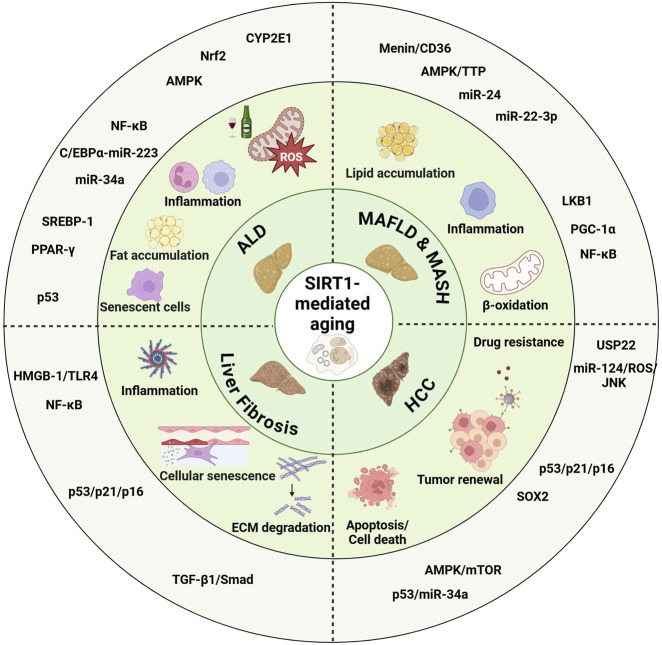
SIRT1 regulates aging-related pathways in liver diseases, including alcoholic-associated liver disease (ALD), Metabolic Dysfunction-Associated Steatotic Liver Disease (MASLD), Metabolic Dysfunction-Associated Steatohepatitis (MASH), liver fibrosis, and hepatocellular carcinoma (HCC). SIRT1 modulates key processes such as inflammation, cellular senescence, fat accumulation, oxidative stress, and drug resistance. By influencing these cellular changes, SIRT1 impacts liver disease progression and contributes to therapeutic resistance, highlighting its potential as a therapeutic target. Created with BioRender.com.

## 1 Introduction

Aging is a fundamental biological process that affects the functionality of various organs (Li et al., 2024), including the liver, which is critical for metabolic homeostasis, detoxification, and energy regulation (Liangyou, 2014; Qiao et al., 2023). As the liver ages, it becomes increasingly susceptible to diseases such as MASLD, MASH, ALD, fibrosis, cirrhosis, and hepatocellular carcinoma (HCC) (Qiao et al., 2023; Maeso-Díaz and Gracia-Sancho, 2020; Baiocchi et al., 2021). Liver disease represents a substantial global health challenge, causing approximately 2 million deaths annually and contributing to nearly 4% of global mortality (Devarbhavi et al., 2023). The rising prevalence of liver conditions, particularly in aging populations (Devarbhavi et al., 2023; Agarwal, 2021), is driven by molecular and cellular changes, including oxidative stress, mitochondrial dysfunction, chronic inflammation, and impaired regenerative capacity (Agarwal, 2021; Hunt et al., 2019; Sanfeliu-Redondo et al., 2024; Lee et al., 2019). Understanding the mechanisms underlying liver aging is essential for developing targeted therapeutic strategies to mitigate these liver-related diseases.

One promising target in aging-related liver diseases is the sirtuin family of proteins, which have gained considerable attention for their roles in metabolism and aging. Among the seven sirtuins, SIRT1, a nicotinamide adenine dinucleotide (NAD⁺)-dependent deacetylase, is the most extensively studied and has emerged as a central regulator of senescence-related liver changes (Wu et al., 2022). SIRT1 influences critical hepatic processes by deacetylating key transcription factors and enzymes involved in glucose and lipid metabolism, inflammation, DNA repair, mitochondrial function, cell proliferation, and cellular senescence (Wu et al., 2022; Chen et al., 2020; Begum et al., 2021; Cetrullo et al., 2015; Chang and Guarente, 2014). Numerous studies have demonstrated that activating SIRT1 can counteract many of the detrimental effects of aging on the liver by preserving liver function, reducing cellular senescence, enhancing stress resistance, and promoting autophagy, which collectively protects the liver from injury and fibrosis (Xu et al., 2020; Guo et al., 2021; Dai et al., 2024). Furthermore, SIRT1 activation has been linked to extended life span and improved health span in various organisms, underscoring its therapeutic potential in age-related liver diseases (Hubbard and Sinclair, 2014; Mendelsohn and Larrick, 2017). In addition to the natural aging process, environmental stressors such as exposure to toxic chemicals and alcohol consumption accelerate liver aging and contribute to disease progression. In this context, upregulating SIRT1 has also been shown to mitigate arsenic-induced mitochondrial dysfunction and cellular senescence, highlighting SIRT1’s crucial role in maintaining liver health under stress (Wang et al., 2024).

This review aims to provide a comprehensive overview of the role of SIRT1-mediated aging in liver diseases and its impact on liver disease progression. By regulating pathways associated with aging, SIRT1 affects the development of various liver conditions, including liver fibrosis, ALD, MAFLD, MASH, and HCC. Understanding the precise mechanisms by which SIRT1 influences these diseases could pave the way for developing SIRT1-targeted therapeutic strategies. Such interventions may offer the potential to slow hepatic aging, prevent or treat aging-related liver diseases, and improve overall liver health and longevity.

## 2 Structure and biological function of SIRT1

SIRT1 is a critical member of the sirtuin family, renowned for its reliance on NAD⁺ as a substrate for its enzymatic activity (Shen et al., 2023; Davenport et al., 2014; Liu et al., 2022a). Each deacetylation cycle of SIRT1 consumes one NAD⁺ molecule, producing acetyl-ADP-ribose (1′-O-acetyl-ADP-ribose or 2′- and 3′-O-acetyl-ADP-ribose) and nicotinamide as byproducts (Liu et al., 2022a; Tang, 2016). Structurally, SIRT1 consists of 747 amino acids and is composed of four key domains: the N-terminal, catalytic core, allosteric site, and C-terminal, with the catalytic core and allosteric site forming the active center of the enzyme (Liu et al., 2022a). The catalytic domain, characterized by the canonical sirtuin fold, is central to SIRT1’s enzymatic function. This domain interacts with the C-terminal regulatory segment (CTR), a crucial modulator of SIRT1’s activity. The CTR forms a β-hairpin structure that complements the β-sheet of the NAD⁺-binding domain, covering a hydrophobic surface that is highly conserved across sirtuins. This interaction between the CTR and catalytic domain stabilizes the enzyme in its closed conformation when bound to NAD⁺ and its substrate, demonstrating SIRT1’s dynamic regulation and adaptability in interacting with various substrates and cofactors (Davenport et al., 2014). SIRT1 is mainly located in the nucleus of most cell types, where it deacetylates crucial transcription factors, influencing gene expression (Liu et al., 2022a). However, SIRT1 also possesses both nuclear localization signals and nuclear export signals, enabling it to shuttle between the cytoplasm and the nucleus. This ability to translocate suggests that SIRT1 can also exert its influence outside the nucleus, particularly at the mitochondria, where it is implicated in regulating mitochondrial metabolism and function (Tang, 2016).

Sirtuins, including SIRT1, are highly conserved across species, reflecting their evolutionary importance in regulating aging, longevity, and metabolic responses (Varghese et al., 2023). Each isoform has specialized functions, with SIRT1 extensively studied for its roles in aging and metabolic diseases. While other sirtuins, such as SIRT2, SIRT3, and SIRT6, contribute to processes like chromatin remodeling, oxidative stress regulation, and DNA repair, this review focuses on SIRT1 due to its critical involvement in cellular mechanisms, particularly in liver diseases associated with aging (Wu et al., 2022). Through deacetylating a wide range of substrates, including both histone and non-histone proteins, SIRT1 modulates various physiological processes including glucose and lipid metabolism, DNA repair and genomic stability, inflammation and immune response. Key substrates include transcription factors such as nuclear factor kappa-light-chain-enhancer of activated B cells (NF-κB), tumor protein 53 (p53), peroxisome proliferator-activated receptor gamma coactivator 1-alpha (PGC-1α), and nuclear factor erythroid 2-related factor 2 (Nrf2), which play integral roles in maintaining cellular homeostasis (da Cunha and Arruda, 2017). By targeting these substrates, SIRT1 regulates processes such as telomere integrity, epigenetic regulation, intercellular communication, inflammation, and mitochondrial function—all of which are essential in mitigating cellular senescence in the liver (Mohammed et al., 2021). The extensive range of physiological functions regulated by SIRT1 highlights its critical role in cellular health and positions it as a potential therapeutic target for age-related diseases and metabolic disorders. As the understanding of the structural and functional dynamics of SIRT1 continues to evolve, its relevance in disease prevention and treatment is becoming increasingly apparent, particularly in the context of liver diseases associated with aging and metabolic dysfunction.

## 3 The regulatory role of SIRT1 in aging

### 3.1 SIRT1’s role in maintaining telomere integrity

Telomeres, the protective caps at the ends of chromosomes, are essential for maintaining genomic stability and preventing cellular senescence (Stock and Liu, 2021). As cells age, telomere shortening becomes a hallmark of aging, closely associated with stem cell decline, fibrotic disorders, and premature aging (Amano et al., 2019). SIRT1 has been evident to preserve telomere integrity by delaying age-related telomere shortening and promoting DNA repair (Lee et al., 2019). Specifically, SIRT1 directly interacts with telomerase reverse transcriptase (TERT), regulates TERT nuclear localization and protein stability, and modulates TERT gene expression through Forkhead Box O3(FOXO3a)/c-MYC pathway, thereby enhancing telomerase activity and stabilizing telomere length (Lee et al., 2024; Cui et al., 2023). Further research using telomerase knockout mice revealed that telomere shortening triggers a p53-dependent repression of SIRT1 through microRNA-mediated post-transcriptional regulation, identifying SIRT1 as a downstream target of dysfunctional telomeres. Therefore, enhancing SIRT1 activity can stabilize telomeres, alleviating telomere-related disorders (Amano et al., 2019). For example, administration of nicotinamide mononucleotide, a precursor of NAD⁺ and an activator of SIRT1, has been shown to enhance SIRT1 activity, preserve telomere length, reduce DNA damage, improve mitochondrial function (Stock and Liu, 2021). Activation of NAD⁺/SIRT1 pathway is involved in inhibiting senescence and improve hepatotoxicity (Cheng et al., 2024; Lei et al., 2024). Conversely, downregulation of SIRT1 is associated with telomere dysfunction, as seen in HCC, where reduced expression of TERT and PTOP, a component of the shelterin complex, is observed (Chen et al., 2011). This dysfunction leads to cellular senescence or apoptosis, emphasizing SIRT1’s critical role in maintaining telomere stability and preventing tumorigenesis. Given that telomerase function plays a significant role in HSCs activation and SIRT1 is capable of influencing the activation state of these cells, it follows that SIRT1 may mitigate cell senescence and inhibit the phenotypic transformation of HSC into pro-fibrotic cells (Sun et al., 2023; Ran et al., 2024). This suggests a potential role of SIRT1-telomerase-HSC axis in the occurrence and development of liver fibrosis, warranting further investigation.

### 3.2 SIRT1’s role in epigenetic regulation

SIRT1 is a critical regulator of chromatin structure and gene expression, influencing both histone and non-histone proteins. In liver diseases, SIRT1-dependent epigenetic regulation plays a central role in modulating cellular metabolism, senescence, and disease progression through mechanisms such as DNA methylation, histone modifications, and interactions with microRNAs (miRNAs). As a key transcription factor, sex-determining region Y-box 2 (SOX2) plays an important role in maintaining the self-renewal ability of stem cells, regulating liver cell differentiation and tissue regeneration. Moreover, SIRT1 regulates the transcription of SOX2 through DNA methylation, leading to hypermethylation of the SOX2 promoter, which affects the self-renewal and tumorigenicity of liver cancer stem cells (CSCs), highlighting SIRT1’s vital role in cancer progression (Liu et al., 2016). Further research has shown that liver proliferation and cancer progression are driven by the downregulation of SIRT1 and its downstream effector, PGC-1α, which is suppressed by the CCAAT/enhancer binding protein β-histone deacetylase 1 complex (Jin et al., 2013). Moreover, dietary interventions such as fasting and resveratrol supplementation have been found to enhance SIRT1 activity, influencing transcription factors such as Forkhead box O3 (FOXO3) and Forkhead box O1 (FOXO1), which improve superoxide dismutase (SOD) activity and reverse oxidative stress-induced acetylation, contributing to liver protection (Hardiany et al., 2023; Das et al., 2016). SIRT1 also interacts with non-coding RNAs, such as miRNAs and long non-coding RNAs, which regulate liver function and disease progression, including miR-34a-mediated repression of SIRT1 in senescence (Ghafouri-Fard et al., 2023; Yang et al., 2024a; Wan et al., 2016).

### 3.3 SIRT1’s role in cellular senescence

Cellular senescence is a key driver of aging and liver disease progression (He et al., 2024; Ge et al., 2023). It is characterized by irreversible cell cycle arrest, telomere shortening, and the secretion of pro-inflammatory factors known as the senescence-associated secretory phenotype (SASP) (Xu et al., 2020; Jiang and Loayza-Puch, 2022). Pro-inflammatory cytokines and chemokines are key players in the function of cell senescence. They can induce inflammation, recruit immune cells, and promote further senescence in neighboring cells (Gwak et al., 2025; Deng et al., 2025; Broman et al., 2024). SASP factors include a variety of cytokines such as interleukin-6 (IL-6) and tumor necrosis factor-α (TNF-α), chemokines such as C-X-C motif chemokine ligand-1 (CXCL1) and CXCL8, growth factors such as vascular endothelial growth factor (VEGF), and proteases such as matrix metalloproteinase (MMPs) (Du et al., 2024). SIRT1 can modulate the SASP by deacetylating and regulating the activity of transcription factors and proteins involved in the production of these factors (Zhao et al., 2019a). For example, SIRT1 can inhibit the activity of NF-κB, a key regulator of pro-inflammatory cytokines, thereby reducing the production and secretion of SASP factors (Chen et al., 2024). In the liver, senescence diminishes regenerative capacity, activates anti-apoptotic pathways, and accelerates the progression of chronic liver conditions such as fibrosis and HCC (Qiao et al., 2023; Lauréline et al., 2021). With age, SIRT1 levels naturally decline, worsening senescence-related dysfunctions such as impaired autophagy, heightened oxidative stress and disrupted cellular metabolism (Qiao et al., 2023; Xu et al., 2020). Enhancing SIRT1 activity has been shown to reverse stress-induced senescence and stimulate liver cell proliferation. For instance, interventions such as Trans-cinnamaldehyde (Rajamani et al., 2015) and carbon monoxide (Park et al., 2020) have demonstrated the ability to reduce SASP factors, boost SIRT1 activity, and delay cellular aging. Additionally, tumor necrosis factor-like weak inducer of apoptosis modulates senescence through SIRT1, reducing senescence-associated β-galactosidase (SA-β-Gal) activity, a well-known marker of senescence (Zhang et al., 2017). SIRT1’s regulatory role in cellular senescence is linked to its ability to target key proteins involved in the senescence pathway, including p53, cyclin-dependent kinase inhibitor 1 (p21), and cyclin-dependent kinase inhibitor 2A (p16) (Zhang et al., 2022). This relationship is further supported by the effects of metformin, a widely used antidiabetic drug that influences SIRT1 and p53 acetylation, helping to balance senescence and apoptosis (Yi et al., 2013).

### 3.4 SIRT1’s role in altered intercellular communication and inflammation

Aging impairs intercellular communication within the liver, especially among hepatocytes, hepatic stellate cells (HSCs), Kupffer cells, and liver sinusoidal endothelial cells (LSECs) (Wan et al., 2022). This breakdown in signaling contributes to fibrosis and chronic inflammation (Wan et al., 2022; Mcdaniel et al., 2017). Decreased SIRT1 expression further aggravates these dysfunctions, especially in hepatocytes and HSCs, where SIRT1 deficiency worsens the cellular environment (Ramirez et al., 2017).

Chronic inflammation is a hallmark of aging livers and is closely associated with the development of liver fibrosis, chronic liver disease (CLD), and HCC. Age-related declines in SIRT1 activity trigger inflammatory pathways such as NOD-like receptor protein 3 (NLRP3) and Interleukin-1 beta (IL-1β), exacerbating liver fibrosis (Adjei-Mosi et al., 2023). Restoring SIRT1 activity can suppress these inflammatory responses, reduce fibrosis, and maintain liver homeostasis. The glycosylation modification of SIRT1 affects its stability, activity and interaction with other proteins, thereby regulating the function and mechanism of SIRT1 in the cell. In the liver, aberrant glycosylation of SIRT1 has been linked to mitochondrial dysfunction and hepatic inflammation, emphasizing the importance of SIRT1 in preventing oxidative stress and maintaining mitochondrial function in aging livers (Chattopadhyay et al., 2020). Further research into how SIRT1 glycosylation modulates its activity in liver cells, particularly in the context of metabolic diseases and fibrosis, could offer valuable insights into its role in maintaining liver health.

### 3.5 SIRT1 and mitochondrial dysfunction

**FIGURE 1:**
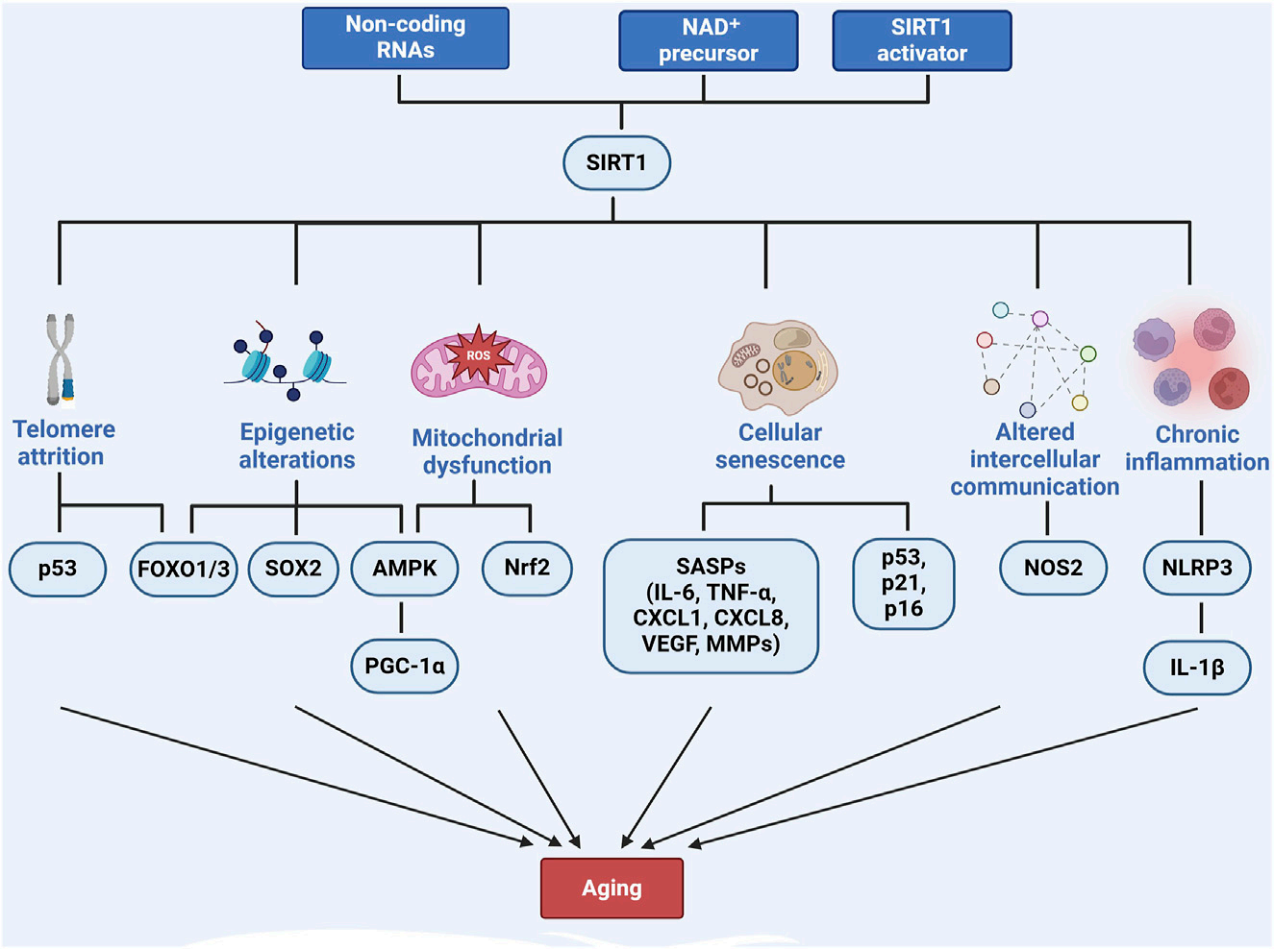
The role of SIRT1 in regulating aging-related pathways. Created with BioRender.com.

Mitochondrial dysfunction is another key feature of aging and contributes significantly to liver diseases such as MASLD and MASH (Song et al., 2023; Gariani, 2017). SIRT1 preserves mitochondrial function by regulating mitochondrial biogenesis, fatty acid oxidation, and reactive oxygen species (ROS) production. As SIRT1 activity declines with age, mitochondrial dysfunction worsens, increasing ROS production and energy conservation and accelerating liver disease progression (Pecher et al., 2020). Interventions such as α-lipoic acid (Yu et al., 2023), epoxyeicosatrienoic acid-agonist (EET-A) (Raffaele et al., 2019) and Mulberry leaf extract (Zhu et al., 2023) have been shown to enhance SIRT1 activity, thereby protecting the liver from mitochondrial dysfunction. SIRT1 activates AMP-activated protein kinase (AMPK), which modulates PGC-1α, promoting mitochondrial biogenesis and reducing oxidative stress (Song et al., 2023; Zhu et al., 2023; Sathyanarayan, 2016). Furthermore, SIRT1 regulates the Nrf2/Kelch-like ECH-associated protein 1 pathway, enhancing antioxidant defenses and further mitigating mitochondrial dysfunction in aging livers (Tang et al., 2014; Dongil et al., 2020). SIRT1 also promotes mitophagy, clears damaged mitochondria, prevents aging-related liver diseases, and improves cellular energy metabolism (Chun et al., 2018) (Figure 1).

## 4 SIRT1-mediated aging in liver disease

### 4.1 Liver fibrosis

Liver fibrosis is characterized by the excessive accumulation of extracellular matrix (ECM) components, primarily collagen type-1, driven by the activation of HSCs in response to chronic liver injury from factors such as viral infections, toxins, or metabolic diseases (Elpek, 2014; Ranjan et al., 2021; Kisseleva and Brenner, 2020). In this process, HSCs transdifferentiate into myofibroblast-like cells, secreting ECM proteins and promoting fibrosis progression, influenced by pro-fibrotic cytokines, oxidative stress, and inflammation (Kisseleva and Brenner, 2020; Hu et al., 2019; Payushina et al., 2021; Nilsson, 2020). SIRT1 has significantly reduced fibrosis by modulating senescence-related pathways that include inflammation, oxidative stress, and ECM remodeling (Dai et al., 2024).

In cholestasis-induced liver disease, SIRT1 activation alleviates mitochondrial dysfunction, reduces apoptosis, and suppresses oxidative stress, all of which contribute to fibrosis and liver aging (Yang et al., 2019; Yu et al., 2016a; Chen et al., 2021; Isaacs-Ten et al., 2022; Jia et al., 2021). This is achieved by upregulating antioxidant defenses, including Nrf2, heme oxygenase-1 (HO-1), and PGC-1α, which enhance mitochondrial function and reduce oxidative damage (Chen et al., 2021; Pan et al., 2022; Liu et al., 2022b). SIRT1 also downregulates pro-senescent proteins such as p53, p21, and p16, protecting against oxidative stress-induced senescence in bile duct cells (Jia et al., 2021; Ma et al., 2021). In addition, SIRT1’s anti-inflammatory effects inhibit key inflammatory pathways central to the fibrotic response, including high mobility group box 1 (HMGB-1)/toll-like receptor 4 (TLR4), NF-κB, peroxisome proliferator-activated receptor alpha (PPARα) and the NLRP3 inflammasome (Mosaoa et al., 2024; Li et al., 2020; Li et al., 2022; Gao et al., 2024). SIRT1 also promotes autophagy through AMPK phosphorylation and increased microtubule-associated protein 1A/1B-light chain 3-II content, offering further protection against fibrosis (Chen et al., 2021; Pan et al., 2022; Abd El Motteleb et al., 2017). In cholestasis liver fibrosis models, SIRT1 inhibits the Transforming Growth Factor Beta 1 (TGF-β1)/Smad pathway, reducing profibrotic markers such as TGF-β, alpha-smooth muscle actin (α-SMA), and collagen I, thereby decreasing HSC activation and epithelial-mesenchymal transition, slowing fibrosis progression (Pan et al., 2022; Abd El Motteleb et al., 2017). However, SIRT1’s role in cholestatic liver injury (CLI) is complex, with both SIRT1 overexpression and depletion showing potential detrimental effects, indicating its dual role in cholestasis (Isaacs-Ten et al., 2022; Blokker et al., 2019).

Aging exacerbates liver fibrosis, as aged mice exhibit more severe fibrosis following hepatotoxin exposure compared to younger mice. This is associated with decreased SIRT1 expression, increased pro-inflammatory responses, NLRP3 activation, and heightened α-SMA content (Adjei-Mosi et al., 2023; Qiu et al., 2023). In aging livers, SIRT1 downregulation causes LSEC dysfunction, worsening liver injury and fibrosis through acetylation of HMGB-1 and activation of the TLR4/protein kinase B (Akt)/endothelial nitric oxide synthase pathway, increasing susceptibility to fibrosis (Dai et al., 2024).

Hepatotoxin-induced fibrosis is also common in liver diseases. In Carbon Tetrachloride (CCl_4_)-treated mice, SIRT1 protein levels significantly decreased, alongside increased expression of cell apoptosis-related proteins. Overexpression of SIRT1 has been shown to reduce TGF-β1-induced expression of myofibroblast markers such as α-SMA and collagen type I alpha (Wu et al., 2015). During the recovery of liver fibrosis, apoptosis of activated stellate cells, along with the reversal of the myofibroblast-like phenotype to a quiescent-like state, plays a pivotal role (Wu et al., 2015). Macrophages assist in fibrosis regression by promoting ECM degradation and inducing myofibroblasts senescence, while LSECs maintain their normal phenotype to facilitate fibrosis resolution. Natural killer cells also participate by targeting and eliminating early-activated HSCs and senescent myofibroblasts (Caligiur et al., 2021). SIRT1-mediated deacetylation preserves LSEC fenestrae, reducing liver fibrogenesis by inhibiting oxidative stress-induced premature senescence (Luo et al., 2021). Moreover, resveratrol (RSV) has been shown to counteract arsenic-induced hepatocyte senescence by restoring SIRT1’s inhibitory effect on p16, thus suppressing SASP-related protein release, reducing fibrotic phenotypes and mitigating liver fibrosis (Ran et al., 2024).

ALD is another major contributor to fibrosis. SIRT1 is associated with miR-34a epigenetic activation and influences the expression of Matrix Metalloproteinase 1 and Matrix Metalloproteinase 2, which are critical in ALD (Meng et al., 2011). miR-34a is regulated through multiple mechanisms, including p53-mediated transcriptional activation and TLR4-mediated inflammatory signaling pathways. By directly targeting the 3′-UTR of SIRT1 mRNA, miR-34a inhibits its translation, thereby reducing the expression of SIRT1 protein and thereby affecting the function of SIRT1 in cells (Wan et al., 2023). In aging livers, restoring SIRT1 protein expression using adenovirus-SIRT1 vectors ameliorated chronic ethanol-induced liver injury and fibrosis, suggesting that aging exacerbates alcoholic liver injury via SIRT1 downregulation in both hepatocytes and HSCs (Ramirez et al., 2017). Additionally, carvacrol and cilostazol co-administration was found to ameliorate ethanol-induced liver fibrosis through the SIRT1/Nrf2/HO-1 pathway, exerting antioxidant, anti-inflammatory, and anti-apoptotic effects (Abu-Risha et al., 2023).

Lastly, biliary senescence and hepatic fibrosis are central features of cholangiopathies, such as primary sclerosing cholangitis (PSC). Senescent cholangiocytes exhibit SASP, promoting fibrosis through autocrine and paracrine mechanisms. In Mdr2 (−/−) mice and human PSC samples, p16 and miR-34a were elevated, while SIRT1 levels decreased, highlighting the importance of modulating the TGF-β1/miR-34a/SIRT1 axis in managing PSC-related fibrosis (Kyritsi et al., 2020).

### 4.2 Alcohol-associated liver disease

Alcohol-associated liver disease (ALD) progresses through a series of stages, from alcoholic fatty liver to alcoholic steatohepatitis, cirrhosis, and HCC (Ramirez, 2018). ALD is characterized by lipid accumulation, liver injury, and inflammation, driven primarily by chronic ethanol exposure, a significant cause of morbidity and mortality worldwide (Chen et al., 2018; Li and Zhou, 2017). The pathogenesis of ALD involves multiple interconnected aging-related processes, including oxidative stress, inflammation, and mitochondrial dysfunction, all of which are regulated by SIRT1, a key modulator of cellular stress responses (Ramirez et al., 2017). Aging exacerbates ALD progression, as older individuals are more susceptible to ethanol-induced liver damage due to declining SIRT1 activity (Ramirez et al., 2017). Studies in aged mice exposed to chronic ethanol show increased steatosis, neutrophil infiltration, and fibrosis, correlating with reduced hepatic SIRT1 levels (Yin and Wang, 2017).

SIRT1 mitigates ALD by inhibiting oxidative stress, a major contributor to ethanol-induced cellular senescence in the liver. Chronic ethanol consumption generates ROS through alcohol dehydrogenase (ADH) and the cytochrome P450 2E1 (CYP2E1)-dependent microsomal ethanol-oxidizing system (Ramirez, 2018; Li and Zhou, 2017), leading to hepatocellular injury and fibrosis (Kitano et al., 2022). SIRT1 counters oxidative stress by deacetylating transcription factors such as Nrf2, thereby increasing the expression of antioxidant enzymes that reduce ROS production (Nagappan et al., 2020). Additionally, SIRT1 activates histone methyltransferases, which promote histone H3 lysine 9 methylation and suppress ethanol-induced CYP2E1 expression, further reducing ROS accumulation (Kitano et al., 2022). Activation of the hepatic adiponectin-SIRT1-AMPK pathway by SIRT1 activators such as Gomisin N (Nagappan et al., 2018) and RSV (Szkudelski and Szkudelska, 2018) has also been shown to alleviate oxidative stress and reduce liver cell senescence, highlighting the therapeutic potential of SIRT1 in ALD.

Inflammation is another key driver of ethanol-induced liver damage, particularly in aging, where chronic inflammation contributes to ALD progression. Chronic alcohol exposure activates inflammatory pathways such as NF-κB, promoting the production of pro-inflammatory cytokines. SIRT1 inhibits NF-κB by deacetylating its p65 subunit, thereby reducing chronic inflammation (Zhou et al., 2020). SIRT1 also modulates miRNAs involved in inflammation, such as miR-34a, which is elevated in aging and ALD. By suppressing miR-34a, SIRT1 enhances anti-inflammatory responses, prevents macrophage activation, and reduces endothelial cell dysfunction. *In vivo* studies have shown that miR-34a deficiency restores SIRT1 levels, reduces nitric oxide synthase 2 expression, and decreases liver injury markers, emphasizing the importance of the miR-34a-SIRT1 axis in preventing ALD progression (Mcdaniel et al., 2017; Wan et al., 2023).

Lipid metabolism is significantly affected in ALD, contributing to hepatic steatosis. SIRT1 regulates lipid homeostasis by suppressing lipogenic transcription factors such as sterol regulatory element-binding protein 1 (SREBP1), reducing triglyceride synthesis and preventing fat accumulation in the liver (Chen et al., 2018). In contrast, ethanol-mediated inhibition of SIRT1 disrupts the lipid-1 pathway, leading to steatosis through an altered lipid-1β/α ratio (Yin et al., 2014). Additionally, SIRT1 regulates PPARγ acetylation, a major regulator of lipid metabolism, to prevent excessive lipid accumulation. SIRT1 transgenic mice, which show reduced PPARγ acetylation, are protected from ethanol-induced hepatic steatosis, suggesting that modulating PPARγ acetylation through SIRT1 could be an effective therapeutic strategy (Yu et al., 2016b).

Moreover, SIRT1’s role in regulating cellular senescence is vital in the context of ALD. By deacetylating and inactivating senescence-associated proteins such as p53, SIRT1 reduces the senescence of hepatocytes and HSCs, thus mitigating fibrosis. As aging further amplifies the severity of alcoholic liver injury and fibrosis through SIRT1 downregulation in hepatocytes and HSCs, activating SIRT1 presents a promising therapeutic avenue. Studies have demonstrated that SIRT1 activation reduces liver fibrosis and inflammation in response to chronic ethanol exposure (Ramirez et al., 2017).

The importance of SIRT1 in ALD is underscored by research showing that aged individuals with reduced SIRT1 expression exhibit greater susceptibility to alcohol-induced liver injury and fibrosis. The SIRT1-CCAAT-enhancer binding protein alpha-miR-223 axis, particularly in myeloid cells such as neutrophils, is crucial in preventing liver damage. By targeting SIRT1 or other related genes, miR-223 is involved in the regulation of liver inflammatory response, cell apoptosis, fibrosis and other processes. In both aged mice and humans, downregulation of this axis increases sensitivity to ethanol-induced injury (Ren et al., 2022). Additionally, compounds such as acanthoic acid have been shown to activate the liver kinase B1 (LKB1)-AMPK-SIRT1 pathway, protecting against ethanol-induced liver damage by reducing inflammation and regulating lipid metabolism, providing further evidence of SIRT1’s protective role (Yao et al., 2017).

### 4.3 Metabolic dysfunction-associated steatotic liver disease

MASLD is the most prevalent chronic liver condition globally, characterized by excessive fat accumulation in the liver (Gariani, 2017). Aging significantly increases the risk of MASLD and accelerates its progression to MASH due to metabolic dysregulation and the accumulation of senescent cells (Park et al., 2023a).

SIRT1-mediated signaling pathways have been shown to attenuate key pathological features of MASH, including hepatic steatosis, inflammation, fibrosis, and cellular senescence (Cao et al., 2013; Hua et al., 2021). SIRT1 maintains the balance of fatty acid metabolism by inhibiting fatty acid synthesis, promoting fatty acid oxidation and regulating fatty acid transport (Ishizuka et al., 2020), thereby reducing the severity of MASLD and MASH (Hua et al., 2021; Yang et al., 2024b). For example, SIRT1 interacts with menin, a tumor suppressor and transcriptional regulatory protein that controls fatty acid transporter CD36 expression via histone modification. This interaction modulates fatty acid uptake and metabolism in the liver. Menin deficiency in high-fat diet (HFD) models leads to hepatic steatosis, while menin overexpression reduces triglyceride accumulation, suggesting the menin-SIRT1 axis as a potential therapeutic target for hepatic steatosis and age-related metabolic disorders (Cao et al., 2013). SIRT1 also exerts protective effects through the AMPK-SIRT1 pathway, modulating key factors involved in lipid metabolism and inflammation. For instance, the mRNA-binding protein Tristetraprolin (TTP) is activated by metformin through this pathway, inhibiting tumor necrosis factor-α (TNF-α) production and reducing hepatocyte necroptosis (Park et al., 2023b). TTP further promotes lipophagy by downregulating Rheb expression, inhibiting mTOR complex 1, and enhancing the nuclear translocation of transcription factor EB, hence reducing fat accumulation. TTP deficiency accelerates MASLD progression in aging and worsens the SASP, highlighting the interplay between SIRT1, autophagy, and senescence in MASLD (Park et al., 2023a). Moreover, a feed-forward loop involving miR-24, PPARδ, and SREBP1c contributes to the pathogenesis of MASH and liver fibrosis. This loop perpetuates the activation of miR-24 biogenesis, the suppression of the PPARδ signaling pathway, and the activation of the AMPK-SIRT1-NF-κB-TNFα pathway, further driving disease progression (Song et al., 2018).

Oxidative stress and inflammation also exacerbate MASLD with aging. Elderly mice on HFD develop more severe steatohepatitis due to reduced β-oxidation, increased fatty acid synthesis, and impaired lipid secretion. Both aging and HFD consumption decrease SIRT1 expression, leading to lipotoxicity and metabolic steatohepatitis (Ishizuka et al., 2020). SIRT1 modulates pathways beyond its classic targets, such as LKB1, PGC-1α, and NF-κB, and is also involved in autophagy and FGF21 regulation, which influence lipid metabolism and mitochondrial function (Hua et al., 2021). Compounds such as 4-butyl-polyhydroxybenzophenone have been shown to enhance mitochondrial biogenesis and protect against MASLD-related liver injury via the SIRT1-PGC-1α pathway (Song et al., 2023).

The decline in NAD^+^ levels with aging is a critical factor linking aging to MASLD progression. NAD^+^ deficiency exacerbates metabolic dysfunction in aging livers, but supplementation with NAD^+^ precursors, such as nicotinamide riboside, has shown promise in reversing these dysfunctions (Gariani, 2017). Strategies targeting NAD^+^ biosynthesis or inhibiting negative regulators of SIRT1 may offer new therapeutic avenues for managing age-related MASLD and MASH (Zhou et al., 2016).

### 4.4 Hepatocellular carcinoma

Hepatocellular carcinoma (HCC) is one of the most common and deadly malignancies, particularly prevalent in aging populations. Aging increases the risk of HCC by promoting cellular senescence, chronic inflammation, and metabolic dysregulation (Wong et al., 2021; Farcas et al., 2019; Udoh et al., 2022). SIRT1, a key regulator in these processes, plays complex roles in HCC, influencing autophagy, inflammation, metabolic reprogramming, and drug resistance (Al-Bahrani et al., 2015). SIRT1 plays a dual role in hepatocellular carcinoma (HCC), acting both as a tumor promoter and a tumor suppressor, depending on the context.

**FIGURE 2:**
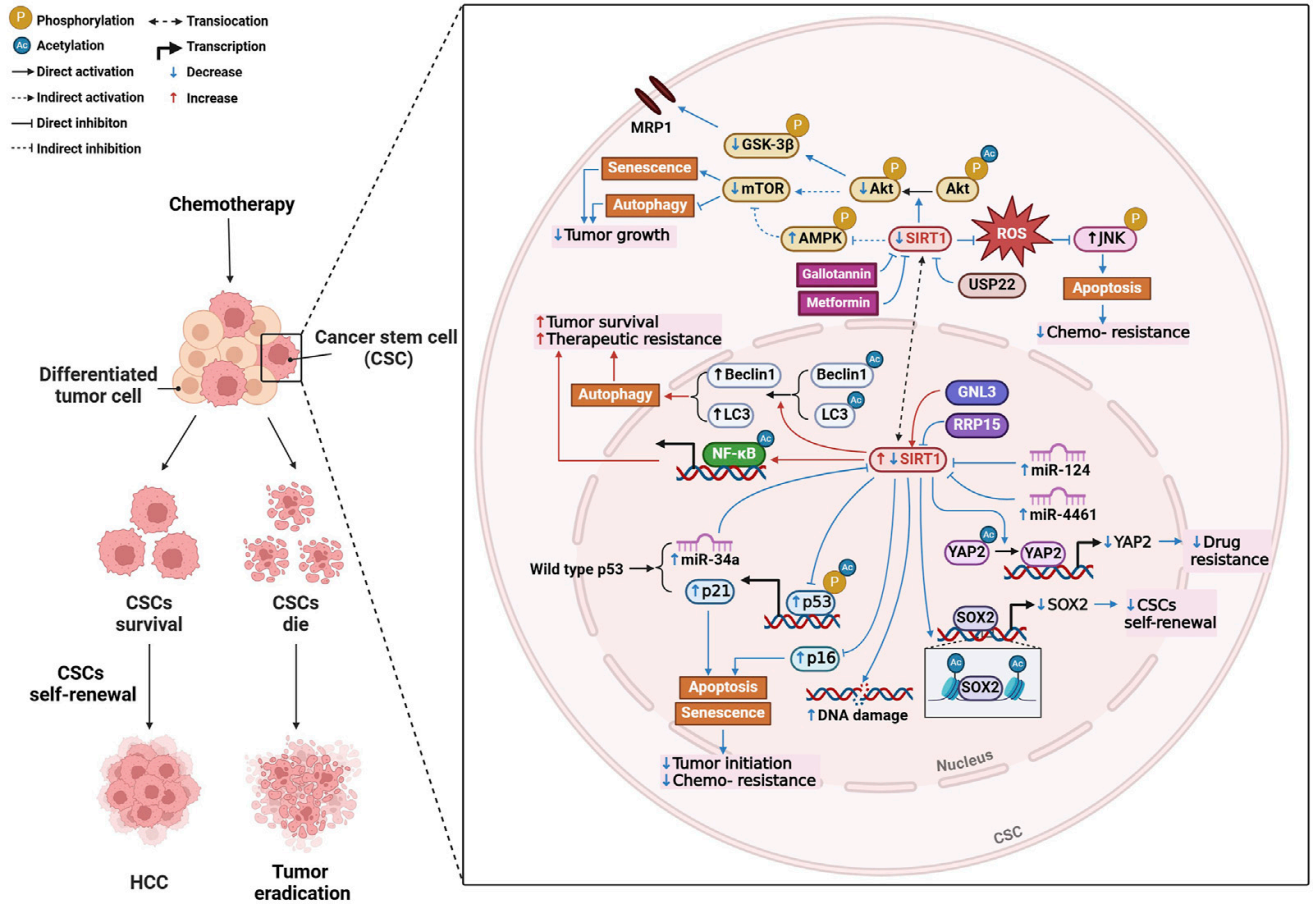
Mechanisms of SIRT1 modulation in hepatocellular carcinoma (HCC). Created with BioRender.com.

Firstly, SIRT1 is essential for maintaining the self-renewal and survival of cancer stem cell (CSCs), which are responsible for tumor initiation, metastasis, and therapeutic resistance (Liu et al., 2016) (Figure 2). High levels of SIRT1 in liver CSCs are associated with poor survival rates in HCC patients, suggesting that its inhibition could be a promising therapeutic strategy (Liu et al., 2016). Further research revealed that inhibition of SIRT1 induces senescence in CSCs by activating the p53-p21 and p16 pathways, thereby reducing tumorigenic potential and enhancing susceptibility to chemotherapy (Wang et al., 2021). It is also found that the loss of miRNAs leads to unchecked SIRT1 activity, contributing to tumor growth, metastasis, and chemotherapy resistance, indicating this is one of the key processes in HCC progression (Farcas et al., 2019). For example, overexpression of miR-4461, which targets SIRT1, inhibits CSC self-renewal and tumorigenesis, improving responses to cisplatin (Yang et al., 2024a). Similarly, miR-124 sensitizes CD133 (+) HCC cells to cisplatin-induced apoptosis by promoting ROS generation and c-Jun N-terminal kinase (JNK) phosphorylation, highlighting the potential of targeting the miR-124/SIRT1/ROS/JNK pathway to overcome drug resistance (Xu et al., 2019). Notably, the p53/miR-34a/SIRT1 positive feedback loop can inhibit cell proliferation and promote apoptosis, providing a potential therapeutic alternative for p53-wild-type HCC patients (Gong et al., 2023). Beyond miRNAs, SIRT1 regulates the transcription of key genes involved in CSC self-renewal, such as SOX2, through epigenetic modifications like histone acetylation and interactions with DNA methyltransferases (Liu et al., 2016). Proteins like GTP-binding nucleolar protein 3 (Cao et al., 2013) and ribosomal RNA processing 15 homolog (Hua et al., 2021), which promote CSC-like features and inhibit senescence through SIRT1, present additional therapeutic targets in treating HCC. Autophagy is another process modulated by SIRT1 that plays a key role in HCC progression and chemoresistance. Targeting SIRT1-mediated autophagy in liver CSCs could reduce tumor progression and impair drug resistance (Wong et al., 2021). SIRT1 deacetylates autophagy-related proteins, such as beclin 1 and microtubule-associated protein 1 light chain 3 (LC3), facilitating the autophagic survival of cancer cells under metabolic stress (Viscomi et al., 2012). In sorafenib-resistant HCC models, elevated SIRT1 enhances autophagy and NF-κB activation, promoting tumor survival and resistance to treatment (Chan et al., 2024).

Conversely, although the upregulation of SIRT1 can promote tumor growth, SIRT1 also plays a role in maintaining the stability of genetic material by reducing DNA damage, especially in aging cells. The loss of SIRT1 leads to increased DNA damage, which in turn promotes the progression of HCC. These interactions suggest that SIRT1 can either promote or suppress tumor development, depending on the context (Varghese et al., 2023).

Pharmacological approaches targeting SIRT1 have shown promise in HCC treatment. For example, gallotannin induces senescence and impairs autophagy by regulating the SIRT1/AMPK axis, decreasing SIRT1 and mTOR expression while enhancing AMPK phosphorylation, leading to tumor growth inhibition (Kwon et al., 2018). Metformin has been explored as an adjuvant therapy for HCC, as low doses promote hepatoma cell senescence by activating the AMPK pathway and inhibiting SIRT1, offering a strategy to reduce tumor growth (Yi et al., 2013; Meng et al., 2023).

Drug resistance, particularly in cisplatin chemotherapy, remains a challenge in HCC treatment. As mentioned above, targeting miRNAs or SIRT1 inhibitors could effectively overcome cisplatin resistance (Xu et al., 2019). Additionally, SIRT1 mediates multidrug resistance through its interaction with ubiquitin-specific peptidase 22 (USP22), which regulates the Akt pathway and multidrug resistance-associated protein 1 expression. Inhibiting the SIRT1/USP22 axis could reverse multidrug resistance and improve chemotherapy efficacy (Ling et al., 2017). SIRT1 also deacetylates yes-associated protein 2 (YAP2), promoting drug resistance by enhancing its transcriptional activation. Inhibiting SIRT1 blocks YAP2 nuclear translocation, improving the chemosensitivity of HCC cells to cisplatin (Mao et al., 2014) (Figure 2).

## 5 Therapeutic strategies targeting SIRT1

The progression of liver diseases accelerates with aging, often leading to end-stage liver disease that necessitates transplantation. Managing chronic liver conditions in the elderly is particularly challenging, underscoring the need for novel therapeutic strategies that address the aging-related decline in liver function (Radonjić et al., 2022). One promising therapeutic target is SIRT1, a regulator of cellular senescence, oxidative stress, and inflammation. By deacetylating key senescence-associated proteins, such as p53, p21, and p16, SIRT1 helps maintain cellular homeostasis and mitigates age-related liver pathologies. Therapeutic strategies targeting SIRT1 could therefore provide significant benefits in slowing liver disease progression by reducing cellular senescence and enhancing tissue repair (Table 1).

**TABLE 1:** Summary of SIRT1 modulators and their aging-related mechanisms in liver diseases.

SIRT1 Modulator	Liver Disease	Model	Outcomes	Mechanisms	Ref
Natural compound					
Apigenin	DILI	CC	↑ autophagy ↓ APAP-induced inflammatory responses and oxidative stress injury	- Regulated the SIRT1-p53 axis, activated the Nrf2 pathway, and inhibited the transcriptional activation of nuclear p65	Zhao et al. (2020)
Celastrol	CLI	Mouse	↑ bile acid metabolism ↓ liver inflammation, apoptosis	- Activated SIRT1, increased FXR signaling and inhibited NF-κB and p53 signaling	Zhao et al. (2019b)
CTXA	LPS-induced liver injury	Mouse	↓ inflammatory responses, cellular senescence	- Increased the expression of Sirt1 and reduced the expression of Ac-FOXO1, acetylated Ac-p53, and acetylated Ac-NF-κB	Lee et al. (2018)
PTE	CLD	Mouse CC	↑ bile metabolism ↓ intrahepatic bile duct mass, hepatic fibrosis	- Regulated the SIRT1-p53 signaling pathway, and the SIRT1-FXR signaling pathway	Ma et al. (2021)
Kaempferitrin	Liver cancer	Mouse CC	↑ apoptosis	- p21/Bcl-2/Caspase 3 signaling pathway	Zhou et al. (2024)
RSV	Liver fibrosis	Mouse	↓ arsenic-induced fibrosis	- Restored the suppression of the senescence protein p16 by SIRT1	Ran et al. (2024)
RSV	H_2_O_2_-induced liver damage	CC	↑ senescence marker protein-30 (SMP30) ↓ H2O2-induced oxidative damage	- Activated AMPK/SIRT1-FOXO1 Signals	Inoue et al. (2023)
PL 1-3	Aging-related liver injury	Mouse	↑ anti-aging ability	- Upregulated Sirt1 expression and downregulated p53, p21, and p16 expression	Li et al. (2023)
PSPC	MASLD	Mouse	↓ HFD-induced hepatocyte apoptosis	- Activated Sirt1 by boosting NAD^+^ level, promoted Sirt1- dependent inhibition of p53-apoptotic pathway and facilitation of Akt survival pathway	Su et al. (2020)
PHGG	Aging-related liver injury	Mouse	↓ oxidative damages probiotic functions	- Significantly regulated the expression of sirtuin 1, FOXO1, p53	Liu et al. (2019)
Synthetic compound					
EET-A	MASLD	Mouse	↓ fatty acid accumulation, liver fibrosis	- An increase in PGC1α-HO-1-PGC1α-mitochondrial signaling	Raffaele et al. (2019)
Empagliflozin	Aging liver	Mouse	↑ lifespan ↓ liver senescence	- Regulated the PI3K/Akt/p21 and AMPK/SIRT1/NF-κB pathways	Long et al. (2024)
GW4064	Aging-related liver disease	Mouse	↑ liver function	- Activated FXR and SHP, inhibited p53 and decreased miR-34a levels, increased SIRT1 levels	Zhen et al. (2016)
Paricalcitol	CLD	Mouse	↑ repair of damaged bile ducts ↓ oxidative stress-induced bile duct epithelial cell senescence	- Upregulated Sirt1 expression, promoted DNA repair, decreased the number of SA-β-gal positive cells, downregulated p53, p21, and p16 proteins expression, and reduced DNA damage	Jia et al. (2021)
PTUPB	MASLD	Mouse CC	↓ liver injury, collagen, lipid accumulation, cellular senescence	- Inhibited the PI3K/Akt/mTOR pathway through Sirt1	Zhang et al. (2022)
RHL	Aging-related induced liver injury	Mouse	↑ mitochondrial function, anti-fibrotic effects ↓ cellular senescence, liver inflammation, oxidative stress	- Improve SOD and GSH-Px activity, reduce MDA levels and modulate the expression of age-associated proteins (Sirt1, p21, p16)	Zhen et al. (2016)
(R)-4′-methylklavuzon	HCC	CC	↑ DNA repair, apoptosis ↓ cell proliferation	- Upregulated SIRT1 protein levels, inhibited CRM1 protein providing increased retention of p53 and RIOK2 protein in the nucleus	Delman et al. (2019)
SRT1720	Impaired liver regeneration	Mouse	↑ liver regeneration ↓ Notch-driven LSEC senescence	- Activated Sirt1 and Notch, upregulated P53, P21, and P16	Duan et al. (2022)
SCIC2.1	HCC	CC	↑ mitochondrial biogenesis ↓ metabolic stress, genotoxic response and senescence	- SCIC2.1-mediated SIRT1 activation, AMPK-p53-PGC1α pathway	Varghese et al. (2023)
EX-527	HCC	CC	↓ cell viability, proliferation, migration, invasion	- Modulated p53 and FoxO1 acetylation, decreased ABC transporters mRNA and protein levels	Ceballos et al. (2021)
Metformin	HCC	CC	Low doses of metformin induced hepatoma cell senescence	- Increased p-AMPK, p-ACC, Ac-p53 and p21 expression, elevated SA-β-gal activity	Yi et al. (2013)
Traditional Chinese medicine formulas					
Livogrit	ALD	CC	Reversal of steatosis through normalization of intra- and extracellular parameters, modulation of lipogenesis, inflammation, autophagy, and β-oxidation	- Upregulated SREBP1c, FAS, PLIN2, TNF-α, NF-kB, and LC3A genes, etc.	Balkrishna et al. (2021)
Qing'E formula	Age-related liver damage	Mouse	↑ liver function	- Elevated gene expression levels of Klotho, Sirt1, FOXO3, PGC-1α, etc.	Zhong et al. (2016)
NAD^+^ precursor					
NAM	Liver fibrosis	Mouse	↑ mitochondrial function ↓ telomere shortening	- Dampens the DNA damage response and p53, upregulated Sirt1 expression	Gariani et al. (2016)
Gene therapy tool					
p16 Vivo-Morpholino	PSC	Mouse	↑ bile metabolism ↓ cholestasis, macrophage infiltration and activation	- TGF-1/miR-34a/Sirt1 axis	Kyritsi et al. (2020)
SIRT1-adenovirus vector	Liver fibrosis	Mouse	↑ HSECs fenestrae ↓ liver fibrosis, oxidative stress-induced premature senescence	- Activated SIRT1 and deacetylated p53	Luo et al. (2021)
Hormone					
DHEA	DILI	Mouse	↑ mitochondrial function ↓ liver inflammation, apoptosis, cellular senescence	- Concomitant upregulation of Bcl-2 and SIRT1-dependent effect	Abd-Ella et al. (2020)
External toxin					
Cd	MASLD	Mouse	↑ liver inflammation, cellular senescence	- The hepatic activation of p53/miR-43a suppressed SIRT1/FXR axis	Alshehri et al. (2021)
Therapeutic agent					
CO	Hepatic ischemia/reperfusion injury	Mouse	↓ liver inflammation, apoptosis	- Increased SIRT1 expression, decreased acetylated p65, p53 levels, and miR-34a expression	Kim et al. (2015)

**Abbreviations**: DILI, Drug-Induced Liver Injury; CC, Cell culture; APAP, Acetaminophen; FXR, Farnesoid X Receptor; Ac-, Acetylated; CTXA, Cudratricusxanthone A; LPS, Lipopolysaccharide; PTE, Pterostilbene; Bcl-2, B-cell lymphoma 2; PSPC, Purple sweet potato color; PHGG, Partially hydrolyzed guar gum; EET-A, Epoxyeicosatrienoic acid-agonist; SHP, Small heterodimer partner; RHL, Rhein lysinate; GSH-Px, Glutathione Peroxidase; MDA, Malondialdehyde; CRM1, Chromosomal Region Maintenance 1; RIOK2, RIO, kinase 2; p-, Phosphorylated; FAS, Fatty Acid Synthase; PLIN2, Perilipin 2; LC3A, Microtubule-associated protein 1 light chain 3 alpha; NAM, Nicotinamide; DHEA, Dehydroepiandrosterone; Cd, Cadmium.

One of the most promising approaches for targeting SIRT1 in liver disease involves using SIRT1 activators, which aim to boost SIRT1’s protective effects. Resveratrol (RSV), a natural compound known for its antioxidant and anti-inflammatory properties, has demonstrated efficacy in reducing liver fibrosis and mitigating cellular senescence by restoring SIRT1 function and downregulating markers such as p16 and p53 (Inoue et al., 2023). By reducing the accumulation of senescent cells, RSV preserves tissue integrity and promotes healthier liver aging. Another synthetic activator, SRT1720, has shown promise in promoting liver regeneration and improving overall liver function, particularly in models of impaired regeneration. SRT1720 activates both SIRT1 and Notch signaling pathways, enhancing tissue repair and reducing cellular senescence (Duan et al., 2022). Additionally, compounds such as PL 1-3 (Li et al., 2023) and Empagliflozin (Long et al., 2024) have garnered attention for their ability to upregulate SIRT1, enhance mitochondrial function, and reduce oxidative stress and senescence markers, thereby mitigating the progression of liver diseases such as MASLD. These activators modulate key pathways, including the phosphoinositide 3-kinase (PI3K)/Akt/p21 and AMPK/SIRT1 axes, emphasizing their therapeutic potential in maintaining liver homeostasis.

In contrast to activators, SIRT1 inhibitors have shown therapeutic promise in certain contexts, particularly in treating liver cancers such as HCC. While SIRT1 typically suppresses senescence, it can promote tumorigenesis in cancers by enabling tumor cells to evade apoptosis. In HCC, SIRT1 inhibition enhances the acetylation of p53 and FOXO1, promoting apoptosis and reducing tumor growth (Ceballos et al., 2018). EX-527, a synthetic SIRT1 inhibitor, has been extensively studied for its ability to suppress tumor cell proliferation by modulating SIRT1-mediated pathways, thereby enhancing apoptosis and decreasing cell viability (Ceballos et al., 2021). Similarly, (R)-4′-methylklavuzon has been shown to enhance SIRT1 expression while promoting apoptosis and inhibiting cell proliferation in HCC cells (Delman et al., 2019). These inhibitors serve as potential therapeutic agents by targeting SIRT1’s role in tumor survival and proliferation, offering a strategy to sensitize cancer cells to treatment.

Gene therapy has emerged as an innovative strategy for directly modulating SIRT1 activity. The use of SIRT1-adenovirus vectors has been shown to increase SIRT1 expression in liver fibrosis models, leading to reduced oxidative stress, decreased fibrosis, and improved liver function (Luo et al., 2021). By modulating SIRT1 expression, these gene therapy tools can limit the accumulation of senescent cells, improving tissue repair and reducing fibrosis progression. Conversely, gene silencing tools such as p16 Vivo-Morpholino can be used to downregulate SIRT1 or other senescence-associated proteins, offering insights into the regulation of liver repair and inflammation. This approach highlights gene therapies’ versatility in upregulating and downregulating key pathways involved in liver disease progression (Kyritsi et al., 2020).

In addition to pharmaceutical approaches, traditional Chinese medicine formulations have shown potential in modulating SIRT1 activity. For example, Livogrit, a tri-herbal Ayurvedic formulation derived from Boerhaviadiffusa L., Phyllanthus niruri L., and Solanum nigrum L., has been shown to reduce oxidative stress and alleviate MASLD -induced liver steatosis (Balkrishna et al., 2021). Similarly, the Qing'E formula, a traditional prescription used since the Song dynasty, has demonstrated potential in modulating SIRT1 activity and reducing inflammation and oxidative stress in liver diseases (Zhong et al., 2016).

Beyond these formulations, various other SIRT1 modulators have shown potential in treating various liver diseases, as summarized in Table 1. These modulators influence critical processes such as oxidative stress, inflammation, cellular senescence, and metabolism, providing diverse treatment options for liver disease management.

## 6 Discussion

Aging significantly affects liver function, increasing susceptibility to diseases such as MASLD, MASH, ALD, fibrosis, and HCC. These conditions are exacerbated by molecular changes such as oxidative stress, mitochondrial dysfunction, and chronic inflammation. As a member of the Sirtuin family, SIRT1 has emerged as a key therapeutic target for aging-related liver diseases. Through its extensive deacetylation of histones and non-histone proteins, SIRT1 influences various intracellular processes. The key target proteins of SIRT1, and their associated functions in liver pathologies are summarized in Table 2 which provides a comprehensive overview of the molecular pathways. Research has demonstrated that SIRT1 is critical in maintaining liver health by preserving telomere integrity, delaying cellular senescence, regulating epigenetic and mitochondrial functions, and suppressing inflammation. SIRT1 activators have demonstrated therapeutic potential in mitigating fibrosis, reducing oxidative stress, and preventing liver injury, while SIRT1 inhibitors show promise in cancer therapy by inducing apoptosis in HCC. Gene therapy and nutritional interventions further enhance the potential for modulating SIRT1 activity in both preventative and therapeutic strategies (Graphical abstract).

**TABLE 2:** SIRT1 target protein deacetylase activity and functional implications.

Target protein	Deacetylase activity by SIRT1	Function/Effect	Associated liver disease	Ref.
NF-κB (p65)	Deacetylates p65 subunit of NF-κB	Reduces inflammation by inhibiting NF-κB activation, lowering cytokine production, and preventing liver fibrosis	ALD, aging-related liver diseases	Zhou et al. (2020)
p53	Deacetylates p53	Inhibits apoptosis and cellular senescence in hepatocytes and HSCs, reducing liver fibrosis	ALD, MASLD	Ramirez et al. (2017)
PGC-1α	Deacetylates PGC-1α	Promotes mitochondrial biogenesis, fatty acid oxidation, and oxidative metabolism, reducing metabolic dysfunction and hepatic steatosis	MASLD, metabolic dysfunction-related liver diseases	Song et al. (2023)
Nrf2	Deacetylates Nrf2	Enhances antioxidant defense by increasing the expression of antioxidant enzymes, reducing ROS production	ALD	Nagappan et al. (2020)
SOX2	Regulates SOX2 expression	Modulates liver CSC self-renewal and tumorigenesis through epigenetic modifications	HCC	Liu et al. (2016)
FOXO3	Deacetylates FOXO3	Promotes stress resistance and longevity, reducing oxidative damage and inflammation in liver tissues	Aging-related liver diseases	Hardiany et al. (2023), Das et al. (2016)
SREBP1	Suppresses SREBP1	Reduces lipid accumulation by inhibiting fatty acid synthesis, helping to alleviate steatosis	MASLD	Chen et al. (2018)
PPARγ	Regulates acetylation of PPARγ	Modulates lipid metabolism by reducing excessive lipid accumulation, preventing hepatic steatosis	ALD, metabolic liver diseases	Yu et al. (2016b)
miR-34a	Modulates miR-34a expression	Enhances anti-inflammatory responses and reduces endothelial dysfunction by regulating miR-34a	ALD, aging-related liver diseases	Mcdaniel et al. (2017), Wan et al. (2023)
Beclin 1	Deacetylates Beclin 1	Facilitates autophagy, promoting cancer cell survival under stress and contributing to chemoresistance	HCC	Viscomi et al. (2012)
LC3	Deacetylates LC3	Increases autophagic activity, contributing to liver CSC survival and chemoresistance in HCC.	HCC	Viscomi et al. (2012)
USP22	Deacetylates USP22	Modulates multidrug resistance and Akt pathway activation, contributing to chemoresistance in HCC.	HCC	Ling et al. (2017)
YAP2	Deacetylates YAP2	Promotes drug resistance in HCC by enhancing YAP2 transcriptional activation, affecting sensitivity to chemotherapy	HCC	Mao et al. (2014)
CYP2E1	Regulates CYP2E1 expression	Suppresses CYP2E1 expression to reduce ROS accumulation and alleviate oxidative stress	ALD	Kitano et al. (2022)
TTP	Activates TTP	Reduces TNF-α production and promotes lipophagy, helping reduce hepatocyte necrosis and steatosis	MASLD, metabolic dysfunction-related liver diseases	Park et al. (2023a) Park et al. (2023b)
Menin	Regulates menin-SIRT1 interaction	Modulates fatty acid transporter CD36 expression and fatty acid metabolism, helping to reduce hepatic steatosis	MASLD	Cao et al. (2013)

Although the protective role of SIRT1 in liver diseases is evident, its precise mechanisms of action remain to be fully understood. Continued research into SIRT1’s regulatory functions will provide essential insights into preventing and treating liver diseases. This review emphasizes SIRT1’s central role in mediating aging and senescence and its beneficial effects on liver health. Future studies should aim to optimize SIRT1 modulators and evaluate their potential in preclinical and clinical settings, offering promising avenues for the treatment of age-related liver conditions.

## Glossary

SIRT1: Silent information regulator 1
MASLD: Metabolic Dysfunction-Associated Steatotic Liver Disease
MASH: Metabolic Dysfunction-Associated Steatohepatitis
ALD: alcoholic-associated liver disease
HCC: hepatocellular carcinoma
NAD⁺: nicotinamide adenine dinucleotide
CTR: C-terminal regulatory segment
NF-κB: nuclear factor kappa-light-chain-enhancer of activated B cells
p53: tumor protein 53
PGC-1α: peroxisome proliferator-activated receptor gamma coactivator 1-alpha
Nrf2: nuclear factor erythroid 2-related factor 2
miRNAs: microRNAs
SOX2: sex determining region Y-box 2
CSCs: cancer stem cells
FOXO3: Forkhead box O3
FOXO1: Forkhead box O1
SOD: superoxide dismutase
SASP: senescence-associated secretory phenotype
SA-β: senescence-associated β
-Gal: -galactosidase
p21: cyclin-dependent kinase inhibitor 1
p16: cyclin-dependent kinase inhibitor 2A
HSCs: hepatic stellate cells
LSECs: liver sinusoidal endothelial cells
CLD: chronic liver disease
NLRP3: NOD-like receptor protein 3
IL-1β: Interleukin-1, beta
ROS: reactive oxygen species
EET-A: epoxyeicosatrienoic acid-agonist
AMPK: AMP-activated protein kinase
ECM: extracellular matrix
HO-1: heme oxygenase-1
HMGB-1: high mobility group box 1
TLR4: toll-like receptor 4
PPAR: peroxisome proliferator-activated receptor
TGF-β1: transforming growth factor beta 1
α-SMA: alpha-smooth muscle actin
CLI: cholestatic liver injury
Akt: protein kinase B
RSV: resveratrol
PSC: primary sclerosing cholangitis
CYP2E1: cytochrome P450 2E1
SREBP1: sterol regulatory element-binding protein 1
LKB1: liver kinase B1
HFD: high-fat diet
TTP: Tristetraprolin
TNF-α: tumor necrosis factor-α
JNK: c-Jun N-terminal kinase
LC3: microtubule-associated protein 1 light chain 3
USP22: ubiquitin-specific peptidase 22
YAP2: yes-associated protein 2
PI3K: phosphoinositide 3-kinase
